# Sepsis research: Heterogeneity as a foundation rather than an afterthought

**DOI:** 10.1016/j.xgen.2024.100608

**Published:** 2024-07-10

**Authors:** Timothy H. Ciesielski

**Affiliations:** 1 Department of Population and Quantitative Health Sciences, Case Western Reserve University School of Medicine, 10900 Euclid Avenue, Cleveland, OH 44106, USA

## Abstract

Our understanding of sepsis has been hampered by the implicit assumption that sepsis is a homogeneous disease. In this issue of *Cell Genomics*, Burnham et al.^1^ have started to characterize the genetic variants and regulatory networks that underlie variations in the individual response to sepsis; this may eventually enable targeted intervention development.

## Teaser

Our understanding of sepsis has been hampered by the implicit assumption that sepsis is a homogeneous disease. In this issue of *Cell Genomics*, Burnham et al. have started to characterize the genetic variants and regulatory networks that underlie variations in the individual response to sepsis; this may eventually enable targeted intervention development.

## Main text

Many human diseases are complex and etiologically heterogeneous, including sepsis. Sepsis is a potentially fatal condition that can be described as a dysfunctional immune response to infection.^2^ However, the nature of this dysfunction is not identical in every case. Overall, sepsis appears to include a mix of several different pathophysiologic states that share common features at certain time points. Thus, proactively separating sepsis subtypes may be necessary to achieve more effective clinical interventions. The work of Burnham et al.^1^ provides one example of this approach.

In 2016, Davenport et al.^3^ used a transcriptomic analysis of peripheral blood leukocytes to identify 2 subtypes of sepsis response: sepsis response signature 1 (SRS1) and sepsis response signature 2 (SRS2). SRS1 is an immunosuppressed subgroup with downregulation of human leukocyte antigen (HLA) class II, T cell exhaustion, and endotoxin tolerance and a higher 14-day mortality than SRS2. Further investigations found that these sepsis response types accounted for more transcriptional variation than the source of the infections—in this case, lung or gastrointestinal tract.^4^^,^^5^^,^^6^ Subsequent analyses indicated that SRS1 was linked to dysfunctional production of granulocytes (granulopoiesis) and higher frequencies of ILR2^+^ immature neutrophils.^5^ This was pointing to a pattern where SRS1 responses have worse prognosis, due, in part, to a proliferation of somewhat ineffective immature neutrophils. Burnham et al. then asked, what increases the likelihood of an SRS1-type response? This is important, as the answer might help to develop immunomodulatory therapies and personalized medicine for sepsis.

Burnham et al. hypothesized that multiple genetic and environmental factors act in concert to determine entry into SRS1 response. To tease out the genetic predictors of the host transcriptomic response, they analyzed patients in the UK Genomic Advances in Sepsis (GAinS) study, which consisted of ∼1,400 patients admitted to intensive care units in the United Kingdom. Confining their analysis to common variants to determine the heritability of ever having an SRS1 transcriptomic response, the authors estimated that this trait was 57% heritable, indicating that there are genetic variants contributing to SRS1 response. They then conducted a genome-wide analysis and identified 155 variants mapping to 25 loci. Many of the SRS-associated SNPs identified here have been linked to immune cell counts, lung function, and clotting factors. Five of these SRS1-associated SNPs were involved in *cis*-expression quantitative trait loci (eQTLs). Two of these five signals, *OCEL1* and *NR2F2*, came from the same genomic region, and *NR2F2* is known to suppresses adaptive immune responses.

Burnham et al. found almost 2,000 sepsis eQTL signals that interacted significantly with (1) SRS category, (2) immune cell proportions, or (3) source of sepsis (community-acquired pneumonia or fecal peritonitis) in their models. The authors also looked into co-expression module QTLs (modQTLs), which identified gene networks. Focusing on hub genes (putative driver transcription factors), they identified the *HIF-1* transcription factor complex (*HIF1α* and *ARNT* [*HIF1β*]), which is involved in immune cell metabolism, coagulation, proliferation, apoptosis, and, ultimately, sepsis survival. This analysis also highlighted *CEBPA* and *CEBPB*, which regulate granulopoiesis. These patterns indicate that SRS1 and SRS2 are different and may require distinct therapies.

Overall, this detailed set of analyses prioritizes the characterization of heterogeneity in the development of sepsis by focusing on the role of host genetic variation in determining response type (SRS1 vs. SRS2). In the future, we will need to better understand how environmental factors, such as common viruses,^7^ may impact the development of SRS1 and, thus, poor outcomes in sepsis. Additionally, we need to learn more about how pathogenic microbes, pharmacotherapies, and the sex and age of the patient may play modifying roles in sepsis response types. In this study, the authors did evaluate sex in their eQTL analyses, but they only conducted follow-up on eQTLs that had an effect in the sex-combined analysis. While this certainly is defensible and can circumscribe follow-up, it may mask key patterns. In other words, if the real effects are quite different in males and females for a given eQTL, then the sex-combined effect is less likely to be significant. Thus, by requiring a significant sex-combined eQTL effect prior to looking for interaction, eQTLs with a real effect in only one sex can be removed before you have a chance to evaluate interaction. This is supported by Ciesielski et al.,^8^ who stratified their genome-wide association analysis of late-onset neonatal sepsis by sex and identified separate sex-specific top hits. Many of these hits did not show up as significant effects in the sex-combined analysis. Of course, every restriction, adjustment, and stratification in observational epidemiology creates the opportunity for collider bias.^9^ In other words, stratification, as the authors do here, can create rather than reduce bias, and thus, corroborating convergent evidence with other approaches is still needed.^10^

**Figure 1:**
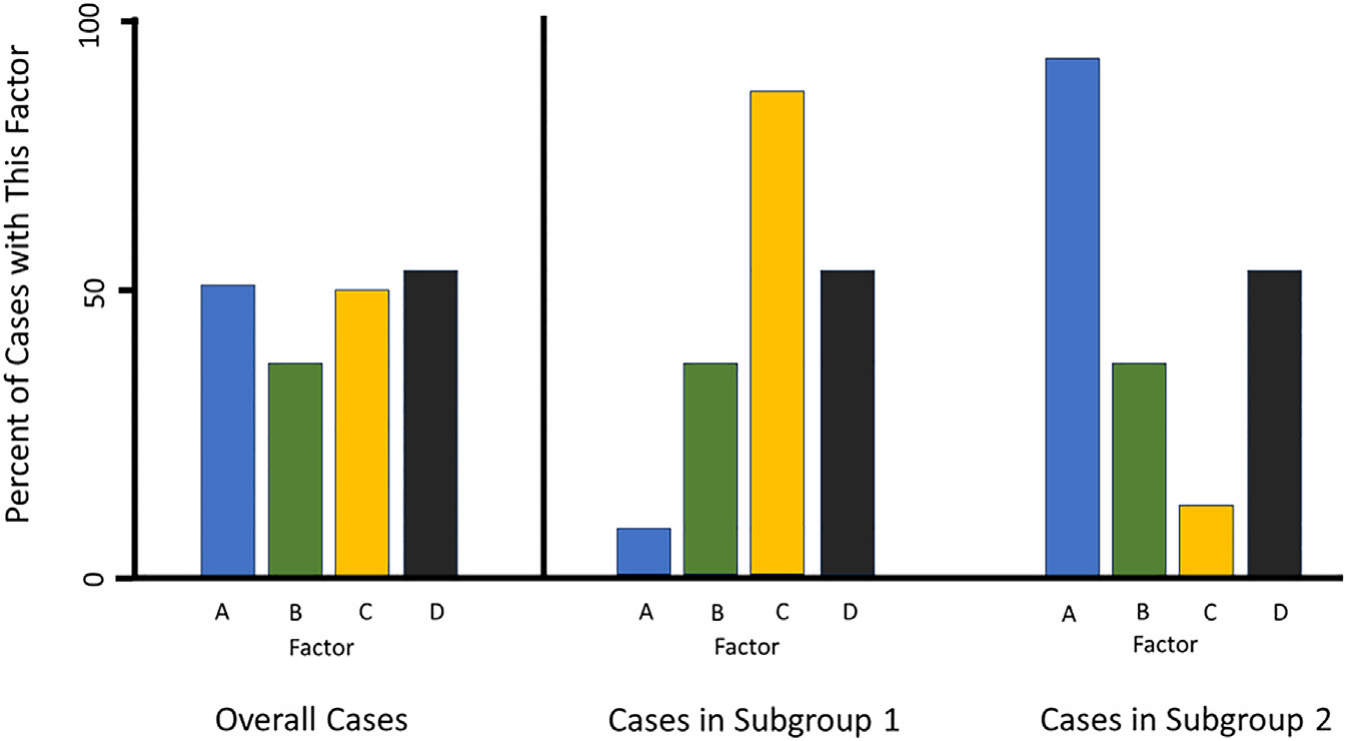
Theoretical example that demonstrates the importance of characterizing heterogeneity If no attempt is made to identify and separate potential etiologic subgroups, then analyses may be misleading. Studying all cases in one group might lead to a dead end or to a tentative focus on factor B. However, if subtypes are identified and studied separately, the roles of factors A and C become obvious and important research leads.

Given that previous efforts have often overlooked sepsis subtypes, I give kudos to Burnham et al. for addressing this in their work. High-power, large-sample-size studies are important, but unaddressed etiologic heterogeneity can undermine their value. Increasing sample size can increase the potential of finding small effects, but if heterogeneity is not characterized, then it can simultaneously lower the probability of finding large effects that only exist in subgroups (Figure 1). Thus, ignoring heterogeneity can impair future research and targeted intervention in infectious disease. Precision medicine in sepsis depends on these efforts to define the subgroups and contexts that matter.
